# Molecular Dynamics Investigation of Wettability Alteration of Quartz Surface under Thermal Recovery Processes

**DOI:** 10.3390/molecules28031162

**Published:** 2023-01-24

**Authors:** Mohammadali Ahmadi, Zhangxin Chen

**Affiliations:** Department of Chemical and Petroleum Engineering, Schulich School of Engineering, University of Calgary, Calgary, AB T2N 1T4, Canada

**Keywords:** heavy oil, contact angle, porous media, molecular dynamics, thermal recovery

## Abstract

One of the primary methods for bitumen and heavy oil recovery is a steam-assisted gravity drainage (SAGD) process. However, the mechanisms related to wettability alteration under the SAGD process still need to be fully understood. In this study, we used MD simulation to evaluate the wettability alteration under a steam injection process for bitumen and heavy oil recovery. Various oil droplets with different asphaltene contents were considered to determine the effect of an asphaltene content on the adsorption of the oil droplets onto quartz surfaces and wettability alteration. Based on the MD simulation outputs, the higher the asphaltene content, the higher the adsorption energy between the bitumen/heavy oil and quartz surfaces due to coulombic interactions. Additionally, the quartz surfaces became more oil-wet at temperatures well beyond the water boiling temperature; however, they were extremely water-wet at ambient conditions. The results of this work provide in-depth information regarding wettability alteration during in situ thermal processes for bitumen and heavy oil recovery. Furthermore, they provide helpful information for optimizing the in situ thermal processes for successful operations.

## 1. Introduction

The world’s primary and vital energy resources are still fossil fuels, including conventional and unconventional oil and gas reservoirs [1,2]. Heavy oil and bitumen have a large share of fossil fuels, but due to their high viscosity, their recovery needs more energy to be injected compared to conventional oil [3,4]. In situ thermal recovery methods, especially steam-assisted gravity drainage (SAGD), are used to extract heavy oil and bitumen [5,6,7]. Although the SAGD process is a typical and mature recovery method for them, its mechanisms related to wettability alteration still need to be fully understood [8].

Modern research uses molecular dynamics (MD) simulations to assess complex interactions from an atomistic perspective, providing in-depth knowledge of static and dynamic features of such phenomena at the atomic level [9,10,11,12,13,14,15,16,17,18,19,20]. For example, Zhong et al. [21] used MD simulations to study the adsorption of crude oil molecules onto water-wet quartz surfaces. They showed that more polarized components can infiltrate a water layer and subsequently adsorb to the quartz surfaces. On the other hand, nonpolar components cannot behave like polar components. They further suggested a two-step adsorption approach in light of their findings. Their concept states that polar oil contents adhere to a rock surface in the first stage, creating space and acting as an anchor for polar and nonpolar elements to attach.

Additionally, adsorption and desorption processes in porous media can be explained by specific mechanisms using MD simulations [22]. MD simulations were used by Du et al. [23] to describe how a cationic surfactant interacts with talc. They demonstrated how the hydrophobicity of a talc surface might be enhanced by a DTAB surfactant, creating a bridge to an edge of the surface. To assess the interactions between the cationic, anionic, and muscovite surfaces of a surfactant combination, Wang et al. [24] employed MD simulation. According to their findings, the key elements in the binding interactions between muscovite and the surfactant mixture are the hydrophobic contacts between surfactant tails and the electrostatic interactions between polar head groups. To grasp how silica nanoparticles’ wettability can improve gas recovery from nanopores, Sepehrnia and Mohammadi [25] used MD simulations. As a result of nanoparticles adhering to a pore wall, their simulated results showed that hydrophobicity increased. It then had an effect of diminishing liquid bridges within nanopores.

To explore the underlying processes influencing the oil recovery from calcite rock during low-salinity water injection, Zhao et al. [26] performed MD simulations. Non-equilibrium MD (NEMD) simulations were also performed to focus on dynamic oil characteristics. They discovered that increasing water’s salt content had no effect on wettability when an environment was oil-wet, but when the environment was partially water-wet, it stimulated wettability toward fully water-wet. They revealed that decane molecules had a propensity to cluster and travel through nanopores as a group. Guo et al. [27] used MD models and laboratory investigations to understand how the quantity and concentration of hydrophilic groups in non-anionic surfactants impacted lignite wettability. According to the findings of their simulations and experiments, they concluded that a linear polyoxy-ethylene ether surfactant has weaker hydrophilic groups than a more effective polyhydroxy surfactant, which allows for a reduction in the hydrophilicity of a lignite surface.

To the best of the authors’ knowledge, no work has been published in the literature using MD simulations to evaluate the effect of asphaltene content on the wettability alteration during in situ thermal recovery processes. Contact angle calculations and adsorption energy of heavy oil droplets onto quartz surfaces were employed to analyze MD trajectories. The subsequent sections explain the MD workflow and details of different simulation configurations. The output of this work provides fundamental information about the impact of heavy oil composition and asphaltene content on the wettability alteration during in situ thermal recovery methods.

## 2. Results and Discussion

**Figure 1:**
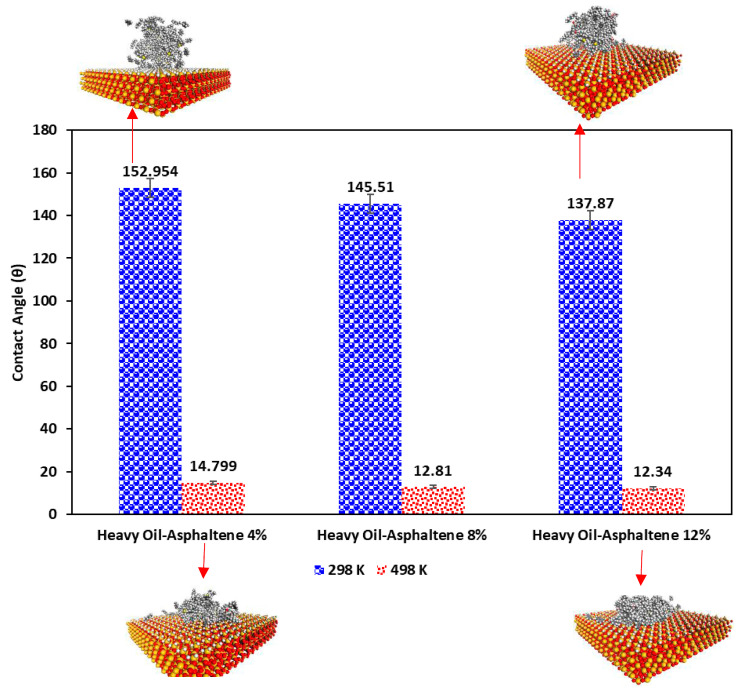
Comparison between the contact angles in each system and different temperatures (for the clarity purposes, water molecules in snapshots are hidden).

Figure 1 compares the contact angles calculated for each system at 298 K and 498 K along with some snapshots of heavy oil droplets on the quartz surface. As shown in this figure, at a temperature of 298 K, the contact angles are well beyond 105°, which reveals a water-wet condition of the quartz surface; however, increasing the asphaltene content of the heavy oil, the contact angle reduced. In other words, increasing the asphaltene content resulted in slight wettability alteration toward oil-wet at 298 K. On the other hand, at a higher temperature (498 K), the contact angle became much less than 75°, which shows a higher tendency of the quartz surface toward oil-wet conditions. Additionally, as illustrated in Figure 1, increasing the asphaltene content can intensify the wetness of the quartz surface toward oil-wet. This observation is attributed to higher adsorption energy between heavy oil droplets and the quartz surface. Our MD simulation outputs are in agreement with experimental observations reported by Naser et al. [28]. It is worth highlighting that the contact angles for heavy oil with 8% and 12% asphaltene contents are similar, which uncovers that at a steam chamber temperature of 498 K, increasing asphaltene content to a specific concentration can reduce the contact angle of the oil significantly, but beyond that concentration, there is no meaningful difference in terms of the contact angle with the quartz surface. The size and structure of asphaltene and resin molecules play a significant role in the colloidal and adsorption behavior of asphaltene molecules in an oil droplet.

The van der Waals interactions are largely determined by the surface area of a molecule and its electronic polarizability. The larger the surface area of a molecule, the more likely it is to have the van der Waals interactions. Electronic polarizability, which describes how easily an electron cloud around an atom can be distorted, is affected by the number of electrons and the size of an atom. Generally, larger molecules have more electrons and are, therefore, more polarizable. The coulombic potential energy is influenced by the polarity of interacting molecules, which can be represented by their dipole moment. The greater the difference in electronegativity between the atoms in a molecule, the larger the dipole moment will be. The presence of heteroatoms can also significantly increase the polarity of a molecule [29,30,31]. Thus, due to the presence of heteroatoms in the molecular structures of asphaltene and resin, these molecules are more polar than saturates and aromatics. It is also worth noting that the numbers of heptane, toluene, and resin molecules for all the three samples are the same and only the number of asphaltene molecules is different. Hence, the changes in both the van der Waals (VdW) and coulombic interactions between heavy oil droplets and a quartz surface mainly come from asphaltene content variations of the three heavy oil samples. Table 1 reports the adsorption energies between the oil droplets and quartz surface for each scenario at different temperatures. It also reports the VdW and coulombic interaction components of the adsorption energy in each scenario. This table illustrates that the adsorption energy between the heavy oil droplets and quartz surface increased by increasing the system’s temperature. Additionally, the coulombic interaction between the heavy oil and quartz is much higher than the van der Waals interaction, which reveals that the polar molecules inside the heavy oil are contributing to the adsorption process of heavy oil droplets onto the quartz surface. In other words, due to the presence of heteroatoms in the molecular structure of asphaltene and resin, these molecules are more polar than saturates and aromatics and interact with the quartz surface via coulombic interactions.

**Figure 2:**
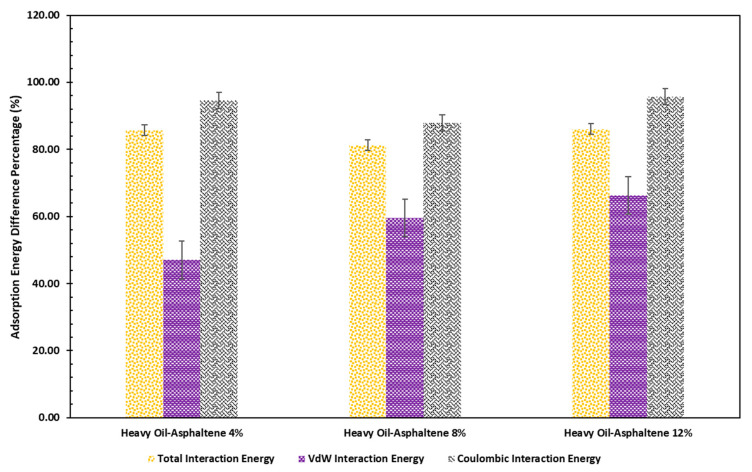
Comparison between the percentage of adsorption energy difference relative to the ambient temperature for each system due to the temperature change from 298 K to 498 K.

Figure 2 demonstrates the percentage of interaction energy difference due to increasing the temperature from 298 K to 498 K. As shown in this figure, increasing the temperature resulted in increasing the adsorption energy by more than 80% due to the extensive contribution of coulombic interactions since asphaltene molecules are polar. Moreover, increasing the asphaltene content can also increase VdW interactions, but the contribution of the VdW interactions is significantly lower than that of the coulombic interactions.

The understanding of the mechanism of wettability alteration under the SAGD process can help to optimize the in situ thermal processes for successful operations. The current study has practical and industrial ramifications in heavy oil recovery, as rock wettability can greatly affect the efficiency of these processes. The results of this study can benefit the optimization of the SAGD process and reservoir simulation in several ways. Firstly, the study provides insights into the mechanisms of wettability alteration during the SAGD process, which can be used to optimize the process and improve recovery efficiency. Secondly, the study found that the asphaltene content in oil droplets affects the adsorption energy between the droplets and the quartz surfaces. This information can be used to optimize the SAGD process for different types of heavy oil reservoirs with varying asphaltene contents. Thirdly, this study revealed that the quartz surfaces became more oil-wet at high temperatures, which can be used as input for reservoir simulation to predict an oil recovery rate and optimize the SAGD process. Lastly, the understanding of the wettability alteration during the SAGD process can be used to design a better SAGD process and improve its oil recovery rate in the field. Additionally, the results of this study are specific to the quartz surfaces, and further research may be needed to understand the wettability alteration on different rock types and fluid types. Overall, this work provides valuable information on the effect of thermal recovery processes on the wettability of quartz surfaces, which can be used to optimize the SAGD process and improve heavy oil recovery.

## 3. Methodology

### 3.1. Force Field and Simulation Initialization

**Figure 3:**
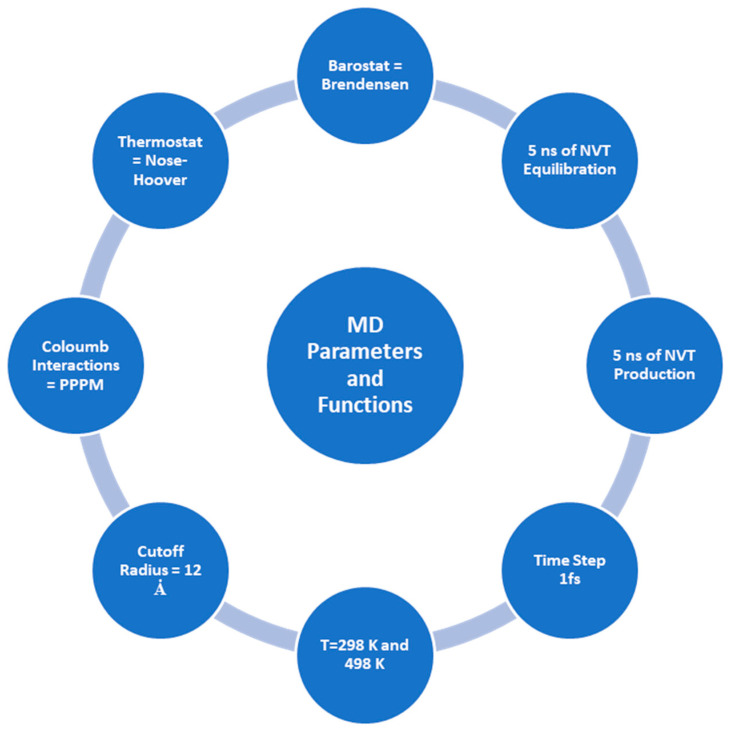
Parameters and MD simulation functions.

**Figure 4:**
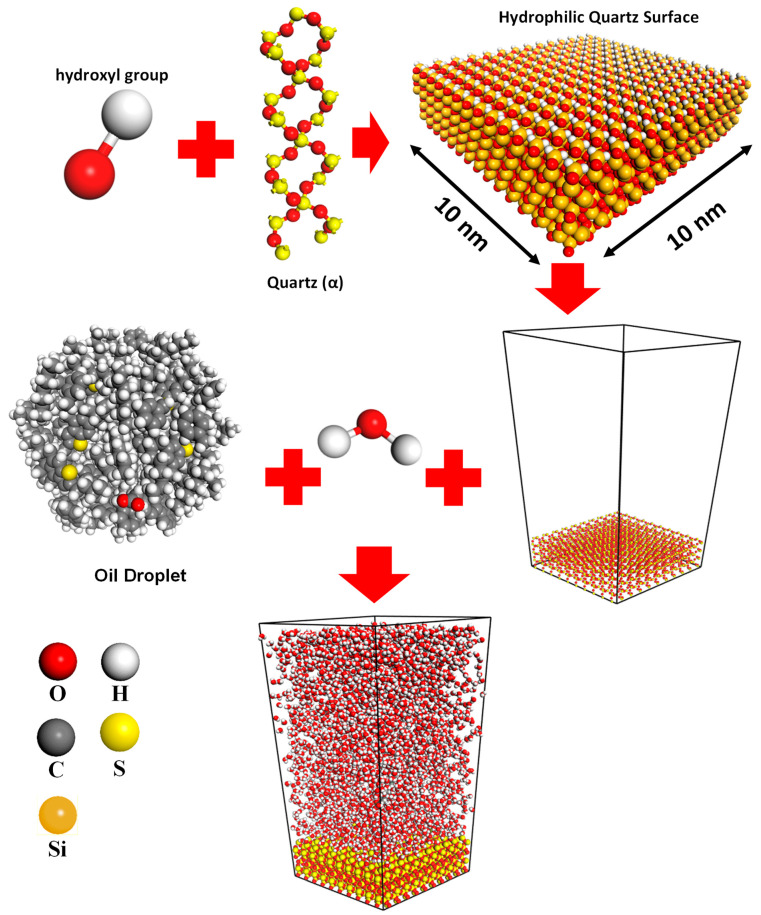
Schematic of simulation box creation to measure a contact angle between a heavy oil droplet on a water-wet quartz.

The Materials Studio software [32] is used to carry out all MD simulations, and the COMPASS III [33,34,35] force field is applied. Various publications in the literature confirm the COMPASS force field’s [33,36,37,38,39,40] ability to represent complicated systems made up of different materials [34,41,42,43,44,45]. For instance, solvation of asphaltene in supercritical water, oil detachment from quartz nanochannels [46,47,48], and carbon dioxide capturing by nanotube–asphalt composite [49]. The typical average temperature in a steam chamber in thermal heavy oil recovery is about 225 °C or 498 K [50,51]. Thus, to evaluate the impact of temperature on the wettability of a quartz surface, two temperatures, 298 K and 498 K, were employed in MD simulations for each system. Table 2 reports the composition of the heavy oil droplets in terms of the numbers of heptane, toluene, resin, and asphaltene molecules. Oil molecules were randomly placed in a 4 × 4 × 4 nm simulation box to create oil droplets. A total of 7000 water molecules were also added to the created oil droplet to generate an oil droplet in a slab filled by water molecules. Then, geometry optimization was performed on the generated oil and water system. The smart method for 2.0 × 10^6^ steps with the energy cutoff of 2.0 × 10^−5^ kcal/mol and cutoff displacement of 10^−5^ Ȧ was employed during the geometry optimization process. Then, 2 × 10^6^ steps of an NPT ensemble with one femtosecond (fs) time step were applied to the simulation box filled by the water molecules and oil droplet to achieve a reasonable density. It is worth noting that this process was performed for each temperature (298 K and 498 K), and the pressure was set to 1 MPa. The generated oil droplet and water molecules were placed onto the quartz surface. In the next step, geometry optimization was applied to the entire simulation box to achieve the minimum energy level. It is worth noting that the geometry optimization setting was similar to the previous stage. In the equilibration stage, we conducted 5 × 10^6^ steps of an NVT ensemble with one fs time step to ensure that the simulation box is equilibrated. Sampling was performed after every 5000 steps of simulation for both the equilibration and production stages. Finally, in the MD production stage, we used 5 × 10^6^ steps of the NVT ensemble with a similar time step to analyze the trajectories and calculate the contact angles and adsorption energies. Figure 3 demonstrates the variables, functions, ensembles, and duration of MD simulations, along with their corresponding values. Figure 4 depicts the schematic of creating an MD simulation box to measure a contact angle between a droplet of heavy oil and a water-wet quartz surface. To figure out the precision of the calculated mean values of contact angle and adsorption energy, error bars as the standard errors were employed, which are calculated as the standard deviation of the sample divided by the square root of the sample size.

### 3.2. Contact Angle Calculations

**Figure 5:**
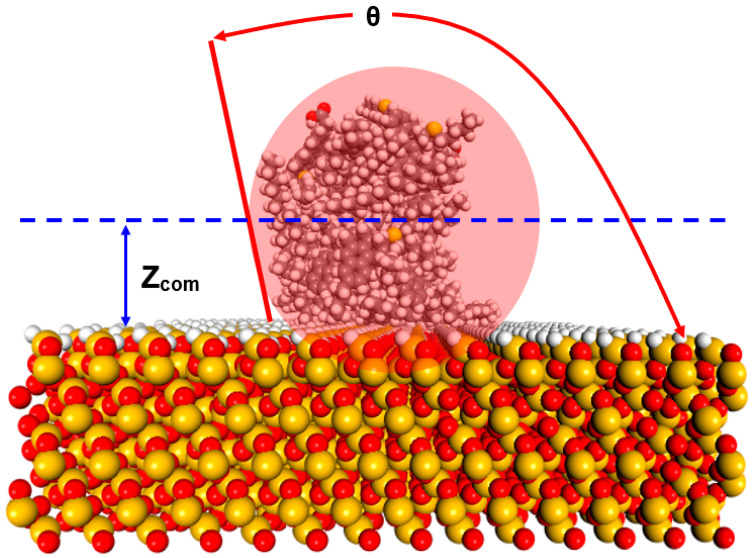
Graphical illustration of the contact angle measurement between heavy oil droplets and a quartz surface.

The wettability of a mineral surface can be quantitatively characterized by a contact angle between a fluid and the mineral surface. Since the parameter of the contact angle cannot be directly obtained in the Materials Studio software, the calculations of the contact angle in this study refer to the Hautman and Klein method [52] through Perl scripting. The Perl Script code is available on request. Moreover, its solution idea is to calculate a microscopic contact angle by comparing the average height of the centroid of an oil droplet <*Z_com_*> with the average height of an ideal spherical droplet intersecting the surface of a calcite. Figure 5 is a schematic diagram of calculating the microscopic contact angle in a molecular simulation. Here, assume that the density of the ideal droplet is homogeneous and equal to the bulk density of oil; that is, the volume of a sphere in the half-space on the surface is equal to the volume of the equivalent water droplet, and the position of the center of mass determines the position of the sphere relative to the plane. Therefore, the angle between the surface of the ideal spherical droplet in Figure 5 and the plane is the contact angle (*θ*) of the water droplet, and the relationship between it and the average height of the droplet centroid <*Z_com_*> can be expressed as follows:

(1) $$\langle Z_{com}\rangle ={\left(2\right)}^{-\frac{4}{3}}R_{o}{\left(\frac{1-cos\theta }{2+cos\theta }\right)}^{\frac{1}{3}}\left(\frac{3+cos\theta }{2+cos\theta }\right)$$

where the average height of the droplet centroid *<Z_com_>* is the height based on the surface of a slab, and the radius of a droplet can be calculated by the total number of molecules (*N*) in the water droplet, which can be expressed as $R_{o}={\left(\frac{3N}{4\pi \rho_{o}}\right)}^{\frac{1}{3}}$. Based on the above method, the microscopic contact angle of water droplets on a calcite surface can be obtained in MS software. It should be noted that a rock surface is predominately water-wet/steam-wet when the contact angle is between 105° and 180°. The rock surface is predominantly oil-wet between 0 and 75 degrees. The contact angle for intermediate-wet rock is between 75° and 105° [28,53].

### 3.3. Adsorption Energy (AE)

Adsorption energy and interaction energy are closely related but different concepts in MD simulations. Adsorption energy is the energy required for a molecule to adhere to a surface and is a measure of the strength of the interaction between the adsorbate (a droplet of heavy oil) and the adsorbent (a quartz surface). If the energy required is negative, it means the adsorbate is stable on the surface, while a positive energy means the adsorbate is not stable. On the other hand, the total potential energy is the sum of the potential energy of each atom in the system, whereas the interaction energy is the sum of the potential energy of each adsorbate–adsorbent pair. In MD simulations, the process of determining the adsorption energy of a molecule on a surface involves a few key steps. First, the initial configuration of a system must be established such that an adsorbate is in contact with the surface of an adsorbent. The total energy of the system, including both the adsorbate and adsorbent, must then be calculated utilizing a chosen force field. Next, the interactions between the adsorbate and the adsorbent must be quantified by summing up the potential energy of each atom in the system. By subtracting the potential energy of the isolated adsorbate and adsorbent from the total energy, the adsorption energy can be determined. The system must be equilibrated for a sufficient period of time before the adsorption energy is calculated to ensure that the system is in thermodynamic equilibrium. After that, a production run should be performed, and the data collected during this time can be processed to calculate the adsorption energy. It is worth noting that this approach assumes that the system is in thermal equilibrium and that the adsorbate–adsorbent interactions are represented by classical potentials, and it may not be able to capture quantum mechanical effects that can influence the adsorption process. To analyze an adsorption process of heavy oil onto a quartz surface, we used the interaction energy between heavy oil droplets and the quartz surface inside a simulation box. The following equation determines our system’s adsorption energy between oil and quartz [54]:

(2) $$E_{Adsorption}=E_{Total}-\left(E_{heavy\;oil}+E_{Silica}\right)$$

where *E_Adsorption_* (kcal/mol) stands for the adsorption energy between the heavy oil droplets and quartz, *E_Total_* (kcal/mol) represents the total energy of the heavy oil droplets and quartz, *E_heavy oil_* (kcal/mol) denotes the energy of the heavy oil droplets inside the system, and *E_Silica_* (kcal/mol) represents the energy of the quartz inside the system [54].

## 4. Conclusions

In this work, we used MD simulations to assess how the wettability of a quartz surface can change under SAGD operation conditions. Additionally, to figure out the effect of asphaltene concentration on the adsorption of heavy oil droplets onto the quartz surface and a wettability change, several heavy oil droplets with varying concentrations of asphaltene were considered. The following conclusions can be drawn from the findings of this study’s MD simulations:

- Increasing the temperature from the ambient temperature (298 K) to a typical steam chamber temperature (498 K) can significantly increase the adsorption energy between heavy oil droplets and the quartz surface. In other words, heavy oil droplets tend to adsorb onto the quartz surface at higher temperatures than ambient conditions.
- Increasing the asphaltene content of the heavy oil can also increase the adsorption energy significantly, and this behavior was intensified at a higher temperature. However, the rate of change of the adsorption energy is not linearly correlated with the asphaltene content of the heavy oil.
- According to the adsorption energy calculations, a coulombic interaction was the main contributor to an adsorption process due to the polarity of the asphaltene and resin molecules since they have heteroatoms in their structures.
- Based on the contact angle calculations, at ambient temperature, a quartz surface tends to be water-wet due to the lower adsorption energy of the heavy oil at that temperature; however, at temperatures well beyond the water boiling temperature, the quartz surface became more oil-wet thanks to higher energy adsorption.
- The results of this study are specific to the quartz surfaces and one type of asphaltene and resin; further research may be needed to understand the wettability alteration on different rock types and various types of asphaltenes and resins.

## Figures and Tables

**Table 1:** Adsorption energy between heavy oil droplets and quartz at 298 K and 498 K.

Temperature (K)	E_Adsorption_ (Kcal/mol)	E_vdw_ (Kcal/mol)	E_coulombic_ (Kcal/mol)
Heavy Oil with 4% Asphaltene			
298	−1308.71	−285.65	−991.33
498	−2430.74	−419.99	−1929.02
Heavy Oil with 8% Asphaltene			
298	−1423.86	−306.18	−1096.85
498	−2580.69	−488.59	−2061.27
Heavy Oil with 12% Asphaltene			
298	−1484.39	−320.82	−1144.11
498	−2762.26	−533.33	−2239.47

**Table 2:** Composition of the heavy oil droplets for each system.

	Heptane	Toluene	Resin	Asphaltene
Heavy Oil ID	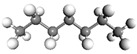	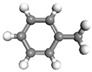	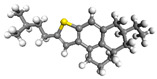	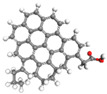
4% Asphaltene	120	120	30	3
8% Asphaltene	120	120	30	5
12% Asphaltene	120	120	30	8

## Data Availability

Data available on request from the authors.
